# Autoimmune Complications of Lymphoproliferative Diseases

**DOI:** 10.1002/hon.70063

**Published:** 2025-06-15

**Authors:** Wilma Barcellini, Bruno Fattizzo

**Affiliations:** ^1^ Hematology Fondazione IRCCS Ca' Granda Ospedale Maggiore Policlinico Milan Italy; ^2^ Department of Oncology and Hemato‐Oncology University of Milan Milan Italy

**Keywords:** autoimmune hemolytic anemia, autoimmune neutropenia, chronic lymphocytic leukemia, immune thrombocytopenia, lymphoproliferative diseases

## Abstract

Peripheral autoimmune cytopenias may complicate a fraction of lymphoproliferative disorders (LPD), particularly chronic lymphocytic leukemia, non‐Hodgkin B‐cell lymphomas, angioimmunoblastic T‐cell lymphoma and large granular lymphocytic leukemia. The most frequent complications are autoimmune hemolytic anemia and immune thrombocytopenia, followed by pure red cell aplasia, autoimmune neutropenia and other systemic/organ specific autoimmune diseases. The latter are less frequently reported and probably underdiagnosed, and include systemic lupus erythematosus, rheumatoid arthritis, Sjögren's syndrome, antiphospholipid syndrome and disorders of the hemostatic system. Therapy is mainly directed at the specific autoimmune complication when it arises in the context of a non‐active LPD. However, autoimmune complications that are refractory to first line therapy usually require an LPD‐directed treatment. Of note, several B‐cell and T cell directed therapies that are used in LPD are also indicated or in trials for primary autoimmune cytopenias, underlying the overlapping pathogenic mechanisms between LPD and autoimmunity.

Peripheral autoimmune cytopenias such as autoimmune hemolytic anemia (AIHA) and immune thrombocytopenia (ITP) have long been known as complications of lymphoproliferative disorders (LPD) [1, 2, 3, 4, 5]. Other autoimmune cytopenias, such as pure red cell aplasia (PRCA), autoimmune neutropenia (AIN) and aplastic anemia (AA) are less frequently reported. Other systemic autoimmune diseases such as systemic lupus erythematosus (SLE), rheumatoid arthritis (RA), Sjögren's syndrome, antiphospholipid syndrome (APS), and organ‐specific autoimmune disorders targeting endocrine glands, liver, and gut, as well as various autoimmune disorders of the hemostatic system are even more rare and probably underdiagnosed [6]. Moreover, there is evidence of an increased presence of autoantibodies in LPD, which may (or may not) anticipate overt disease [7]. Autoimmune complications may greatly impact on the prognosis and therapy of LPD, both for their clinical severity (potentially life‐threatening) or their chronic/relapsing clinical course. Additionally, LPD therapy, such as hematopoietic stem cell transplant (HSCT), chimeric antigen receptor T cells, and checkpoint inhibitors may further trigger autoimmunity [8].

Autoimmunity results from the breakdown of both central and peripheral tolerance against self‐antigens. The former occurs during the maturation of the immune system in the bone marrow and the thymus by negative selection (clonal deletion) of autoreactive immune effectors. Peripheral tolerance is an active and continuous process, mainly acting via induction of an anergy of immune effectors, production of several cytokines, and activation of T regulatory cells (Tregs). Autoimmunity recognizes a genetic background (HLA molecules, mRNA, and polymorphisms of genes encoding for various cytokines) and several environmental factors (virus, bacteria, drugs) via mechanisms known as molecular mimicry or polyclonal activation. Additionally, the “forbidden clone” hypothesis proposed more than 60 years ago has gained a renewed attention, supporting the notion of autoreactivity within the B cell lineage triggered by IL‐10‐ and TGF‐β‐producing Bregs. However, in CLL, autoantibodies are polyclonal high‐affinity IgG produced by non‐malignant self‐reactive B‐cells in 90% of cases, and other mechanisms (imbalance in the T helper 17 (Th17)/T‐regs and Toll‐like receptors) are also reported [5, 9, 10, 11, 12, 13].

AIHA is the most frequent autoimmune complication in chronic lymphocytic leukemia (CLL), being reported in 7%–10% of cases and correlating with advanced disease and high biologic risk. AIHA is frequently described in association with angioimmunoblastic T‐cell lymphoma and marginal zone non‐Hodgkin lymphoma (NHL), while it is rarely reported in Hodgkin lymphoma. Case reports and small series of AIHA have been described in association with large granular lymphocyte leukemia (LGLL), which in turn encompasses several overlapping features with various autoimmune diseases [1, 2, 3, 5, 6].

The diagnosis of AIHA is supported by the presence of anemia (excluding vitamin/iron deficiency, blood loss, renal/liver insufficiency), alteration of hemolytic markers (increased LDH, unconjugated bilirubin, and absolute number of reticulocytes, and reduced haptoglobin), and positive direct antiglobulin test (DAT) or Coombs test [13, 14, 15]. The latter allows to distinguish warm forms (wAIHA) that show a DAT positive for IgG or IgG + C3d at low titer, and cold forms (cold agglutinin disease, CAD) that show DAT positive for C3d, positive autoagglutination at 20°C, and positive serum cold agglutinins with a titer > 1:64. It should be remarked that CAD has been recently included in the WHO classification of LPD since it is characterized by 10%–15% clonal B‐cell bone marrow infiltrate, sometimes difficult to differentiate from other NHLs, and a monoclonal IgM at serum electrophoresis. The term CAS (cold agglutinin syndrome) has been proposed to identify cold AIHAs occurring in the context of other overt LPDs, though the distinction may be difficult. Notably, hemolytic markers may be of uncertain interpretation in LPD‐associated AIHA: LDH may be disproportionally increased and reticulocytosis absent due to bone marrow infiltration or chemotherapy. Moreover, about 5%–10% of AIHAs are negative with the traditional DAT. In these cases, most sophisticated/sensitive DAT methods are required to confirm the diagnosis, such as the microcolumn, solid phase, ELISA, cytometric, and mitogen‐stimulated assays [16, 17]. The latter, which is able to disclose a latent autoimmunity against RBC, was found positive in about 30% of CLL without AIHA [7]. Whether the presence of these autoantibodies may be a predictor for the development of AIHA is still an open question. Finally, it is worth reminding that the DAT may be positive for alloantibodies in recently transfused patients, further complicating the diagnosis [13, 14, 15, 16, 17].

The second most frequent autoimmune complication in LPD is ITP being reported in up to 5% of CLL, sometimes in association with AIHA (Evans syndrome) [1, 2, 5, 6]. ITP has been frequently described in LGLL (up to 20% in some case series), while its frequency is < 1% in NHL and HL. Case reports have been described in Waldenström macroglobulinemia, multiple myeloma, and Castleman disease. The diagnosis may be difficult, generally in exclusion, as anti‐platelets antibodies may be falsely negative (about 50% of sensitivity of the test) or falsely positive for alloantibodies in transfused patients, and bone marrow features may lack increased megakaryocytes [18].

PRCA and AIN are rare (< 1%) either in CLL and lymphomas, due to the rarity of these disorders and the difficult diagnosis [6]. In fact, the former merely relies on marked reduction/absence of erythroid precursors in the bone marrow (sometimes difficult to distinguish from the effects of chemotherapy or bone marrow infiltration). Moreover, PRCA is sometimes difficult to distinguish from congenital Diamond‐Blackfan anemia or other rarer inherited forms, and may be secondary to Parvovirus B19 erythroblastopenia, anti‐erythropoietin antibodies in treated patients, thymoma, other systemic autoimmune diseases, drugs or chemicals [19]. Likewise, the diagnosis of AIN is a diagnosis in exclusion, confirmed by the positivity of anti‐neutrophil antibodies, that have an estimated sensitivity of about 50% [20]. Regarding diagnosis and treatment of this rare cytopenia some recommendations have been recently published in the context of Evans syndrome [21]. At the very end, most diagnoses are made on the basis of an abrupt fall of peripheral counts with or without autoantibody positivity and bone marrow features, and ultimately on the response to immunosuppressive therapy.

Notably, infections are known to be triggers of autoimmunity, as well as complications of specific therapies for LPD. In addition to the above cited Parvovirus B19, it is worth remembering several viruses (HCV, HBV, HIV, CMV, EBV, SARS‐CoV‐2), *Mycoplasma* pneumoniae, *Mycobacterium tuberculosis*, *Treponema pallidum*, and *Brucella* [8]. Moreover, several drugs may be responsible for immune‐mediated cytopenias, including several antibiotics (ceftriaxone, piperacillin, rifampin, nafcillin, erythromycin, ticarcillin, trimethoprim, sulfamethoxazole), and various other drugs (procainamide, quinine, phenacetin, diclofenac, cimetidine, hydrochlorothiazide, chlorpropamide) [8].

Among drugs used in lymphoproliferative disorders, it is worth citing fludarabine, that has been associated with an increased occurrence of autoimmune complications, particularly AIHA, due to the imbalance between Th17 and T‐regs. However, addition of cyclophosphamide and rituximab that kill autoreactive T‐cells markedly reduce this complication [22]. Ibrutinib, through the inhibition of autoantibodies producing B‐cells and restoration of T‐cell homeostasis seems safe, while some case reports of autoimmune diseases have been reported for idelalisib (autoimmune hepatitis, colitis) and venetoclax (AIHA) [5, 6]. Other anti‐cancer treatments associated with immune‐mediated cytopenias include immune checkpoint inhibitors that reactivate T‐cell mediated immunosurveillance against cancer cells, by targeting programmed death receptor 1 and its ligand (PD1 and PDL1) or CTLA‐4. They included nivolumab, followed by pembrolizumab, ipilimumab, and atezolizumab. All cases of AIHA displayed severe hemolysis, with transfusion requirement (80%), high prevalence of DAT negativity (38%), relapse in about 50% of patients, and mortality as high as 17% [8, 23, 24]. Finally, autoimmune cytopenias may complicate HSCT, with most reports regarding AIHA, and a few cases of ITP. They are generally severe and refractory to several therapies. Risk factors include unrelated donor and HLA mismatch, use of cord blood, occurrence of graft‐versus‐host disease, age < 15 years, CMV reactivation, and alemtuzumab use [8, 17].

Therapy of autoimmune cytopenias depends on the timing of their onset, that is before, concomitant or during/after therapy for LPD. In the former, therapy is that of the specific cytopenia and is essentially based on expert consensus and few prospective clinical trials [13, 14, 15, 16, 17]. For warm IgG‐mediated AIHA, first line is based on steroids (usually prednisone at 1 mg/kg day for 3–4 weeks, followed by a slow tapering in about 6 months) with or without intravenous immunoglobulins (0.4 g/kg for 5 days or 1 g/kg for 2 days). Methylprednisolone boli (2–10 mg/kg Day for 3 days) may be considered in patients with acute hemolysis and slow response to steroid therapy. Rituximab (375 mg/sqm weekly for 4 weeks) is widely used as second line in primary AIHA forms [13, 14, 15, 16, 17, 25, 26]. For CAD, steroids are poorly effective, and rituximab is indicated as first line. Plasma exchange may be effective as a temporary measure in both warm and cold forms [13, 14, 15, 16, 17, 27, 28]. The complement inhibitor sutimlimab is highly effective, provided that the diagnosis is still CAD, while it has not formally been investigated in CAS [29, 30, 31]. Sutimlimab requires continuous administration every 2 weeks, and new and long‐acting complement inhibitors are under investigation (riliprubart, pegcetacoplan, Iptacopan) [27, 28, 32]. Supportive treatments include folic acid, transfusions and erythropoietin, particularly in cases with reticulocytopenia, either for bone marrow involvement or previous chemotherapy [33]. For relapsed/refractory cases usually an LPD‐directed therapy is indicated along with careful staging. In particular, in CLL several therapies have been reported effective in refractory cytopenias: alemtuzumab single agent, the combinations ibrutinib‐rituximab, bendamustine‐rituximab, and rituximab–cyclophosphamide‐dexamethasone [34, 35, 36, 37]. Notably, some of the new/experimental treatments for primary AIHAs encompass B‐cell targeting therapies that are currently administered in LPD, such as ibrutinib, rilzabrutinib, zanubrutinib, bortezomib, daratumumab, isatuximab, and parsaclisib [13, 14, 15, 16, 17, 26, 27, 28, 38, 39, 40, 41]. Regarding chemo‐immunotherapy, the association rituximab‐bendamustine and rituximab‐fludarabine have been proposed for refractory CAD patients [15, 27, 28]. As already stated, fludarabine has been associated, as single agent, with a high incidence of AIHA in CLL, while the combination with rituximab seems protective, as well as new molecules (however most trials in LPD excluded DAT‐positive patients). Splenectomy and classic immunosuppressors are usually not recommended in LPD‐secondary cases of AIHA, mostly for the infectious and thromboembolic risks.

Therapy of ITP is analogous to that of primary forms when cytopenia precedes the need for LPD‐specific therapy. Steroids, with or without intravenous immunoglobulins as first line, and rituximab or thrombopoietin receptor agonists as second line are usually administered [18, 42, 43, 44]. As for AIHA, relapsed/refractory ITP cases usually require an LPD‐directed therapy. There is no specific indication for AIN, but careful monitoring and prompt therapy of infections. There is no agreement on prophylactic antibiotics and G‐CSF seems more appropriate in the case of acute/severe infections rather than as continuous administration [20, 21]. Therapy of PRCA is mainly based on steroids and cyclosporine and on the underlying condition (thymoma, systemic autoimmune diseases, B19 parvovirus, avoidance of drugs or chemicals). In PRCA secondary to CLL, rituximab has been used with efficacy. Other treatments include sirolimus, alemtuzumab, bortezomib, or daratumumab [19]. In cases associated with LGLL, the preferred immunosuppressors are methotrexate and cyclophosphamide [45]. As for other LPD‐related autoimmune cytopenias, relapsed/refractory cases require a specific LPD therapy.

Regarding other autoimmune diseases, the most frequent association is reported for T‐LGL disorders. More than 50% of cases have been diagnosed with RA, SLE, Hashimoto thyroiditis, and Sjögren syndrome [6]. Diffuse large B cell lymphoma was reported associated with Hashimoto thyroiditis in 31% and RA in 23% of cases. CLL has been reported associated with the above‐mentioned conditions in about 2% of cases. Some case reports describe CLL‐or lymphoma (mainly marginal zone)‐associated acquired hemophilia, acquired von Willebrand syndrome, and APS. Finally, the rarest complications of LPD, possibly confounding the diagnosis, include vasculitis, polymyositis, systemic sclerosis, mixed connective tissue disease, seronegative spondylarthritis, inflammatory bowel diseases, autoimmune hepatitis, glomerulonephritis, pemphigus, diabetes type 1, and Addison's disease. All these diseases require high expertise both for the diagnosis and therapy [6].

In conclusion, autoimmune cytopenias, mainly AIHA and ITP, may complicate a fraction of LPD, particularly CLL, B‐cell NHL, angioimmunoblastic T‐cell lymphoma, and LGLL, and are generally associated with active disease and poor prognosis. More rare complications include AIN, PRCA, and other systemic or organ specific autoimmune disorders (SLE, RA, Sjögren's syndrome, APS and many other). The diagnosis is quite simple for AIHA and ITP (generally suspected when there is a rapid drop of blood counts and confirmed by autoantibody positivity) while other complications require high awareness and knowledge of possible LPD‐related confounding factors (bone marrow infiltration, therapy, etc.). Therapy is mainly directed at the specific autoimmune complication when it arises in the context of a non‐active LPD. However, autoimmune complications that are refractory to first line therapy usually require an LPD‐directed treatment. Of note, several B‐cell and T cell directed therapies that are used in LPD are also indicated or in trials for primary autoimmune cytopenias, underlying the overlapping pathogenic mechanisms between LPD and autoimmunity.

## Conflicts of Interest

W.B. has received fees for Consultancy/Advisory Board from Agios, Alexion, Biocryst, Incyte, Momenta, Novartis, Roche, SOBI, Sanofi, and research support from Alexion. B.F. has received consultancy or advisory board honoraria and speakers bureau from Agios, Alexion, Apellis, Janssen, Novartis, Roche, Samsung, Sanofi and SOBI.

## Peer Review

The peer review history for this article is available at https://www.webofscience.com/api/gateway/wos/peer-review/10.1002/hon.70063.

## Permission to Reproduce Material From Other Sources

The authors have nothing to report.

## Data Availability

The authors have nothing to report.
